# A multicomponent microemulsion using rational combination strategy improves lung cancer treatment through synergistic effects and deep tumor penetration

**DOI:** 10.1080/10717544.2017.1365394

**Published:** 2017-08-25

**Authors:** Ding Qu, Mengfei Guo, Yue Qin, Lixiang Wang, Bing Zong, Yunyan Chen, Yan Chen

**Affiliations:** a Affiliated Hospital of Integrated Traditional Chinese and Western Medicine, Nanjing University of Chinese Medicine, Nanjing, PR China;; b Jiangsu Province Academy of Traditional Chinese Medicine, Nanjing, PR China;; c Zhenjiang Hospital of Chinese Traditional and Western Medicine, Zhenjiang, PR China

**Keywords:** Combination therapy, anti-lung cancer, multicomponent microemulsion, tumor penetration, cytokines

## Abstract

Previously, we have developed a multicomponent-based microemulsion composed of etoposide, coix seed oil, and ginsenoside Rh2 (ECG-MEs). In this study, our goal was to validate the feasibility of ECG-MEs in lung cancer treatment and explore the mechanism underling the enhanced antitumor efficacy. The optimal weight ratio of ginsenoside Rh2 (G-Rh2) in ECG-MEs was determined as 3% (wt%), that was capable of forming the microemulsion readily with small particle size and high drug encapsulation efficiency. In cellular studies, the intracellular fluorescence of human non-small cell lung cancer (A549) cells treated with fluorescein isothiocyanate-labeled ECG-MEs (FITC/ECG-MEs) was significantly higher than that of various controls, leading to the obviously synergistic anticancer activities in cytotoxicity and *in vitro* cell apoptosis induction. The anticancer efficacy *in vivo* results showed that ECG-MEs markedly inhibited the growth of A549 tumor xenografts, potently induced tumor cells apoptosis, and obviously prolonged the survival time of mice. Of note, the mechanisms of enhanced anticancer efficiency were connected with the small size-mediated deep tumor penetration and increase in serum concentration of T helper 1 (Th1) cytokines. In summary, ECG-MEs exerting the rational drug combination strategy offers a solid evidence for lung cancer treatment, and has a promising potential for clinical application.

## Introduction

Multicomponent-based combination chemotherapy is now receiving the increasingly concerns, due to the complex physiological mechanism of tumors that monotherapy is incapable of modulating various signaling pathways and thereby failure in highly effective antineoplastic treatment (Hanahan & Weinberg, 2000; Kitano, 2007; Miao & Huang, 2015). Rational drug combination can affect multiple targets within the cancer cells, realize the synergetic responses, enhance the therapeutic effects, reduce the systemic toxicity and even overcome drug resistance (Xiong & Lavasanifar, 2011; Duan et al., 2013; Xu et al., 2013). For instance, doxorubicin (DOX) utilized as a potent chemotherapeutic via interfering DNA synthesis of cancer cells gains a significant enhancement on the treatment of non-small cell lung cancer after combination with paclitaxel (PTX) that kills tumor cells through promoting microtubule polymerization (Lv et al., 2014). Benefiting from the fully elucidated mechanisms of antitumor agents underlying activities, the novel strategy of drug combination offers an unparalleled opportunity for curing the cancer. However, the traditional cocktail combination treatments still face substantial issues urgently needed to address, including varying biodistribution/metabolism among various components (Moghimi et al., 2001), poor tumor-specific targeting (Peer et al., 2007), lack of selectivity to tumor from healthy tissues (Medina et al., 2007) and failure to deliver drug to the deep layer of tumor tissues (Endres et al., 2012).

With the profound understanding of molecular biology in cancers and the development on the biomaterials, the emergence of therapeutic nanoparticles provides varying feasible drug carriers to perform combinational administration (Shi et al., 2010). Unlike the free drugs, nanoparticles have a lot of distinct advantages, including solubilization of water-insolvable drugs (Qu et al., 2013), improved tumor cellular uptake (Wang et al., 2011), prolonged blood circulation (Zhao et al., 2016), minimized systemic toxicity (Mo et al., 2014), and enhanced accumulation at tumor sites (Jia et al., 2009). As for the tumor targeting, enormous efforts have been made in design of nano-sized drug delivery system, such as regulation of particle size/inflexibility to achieve a maximized EPR (enhanced permeation and retention) effect and modification with tumor-targeted ligands to acquire a tumor-seeking ability (Li & Huang, 2010; Deng et al., 2017). Unfortunately, clinical trials recently released a mixed report that only less than 5% of nanoparticles in most cases could reach the vicinity of tumor, regardless of that based on the passive targeting or active targeting strategy (Wilhelm et al., 2016; Miller et al., 2017). Two reasons can explain the issues that nanomedicine encountered presently. One is difficult delivery of nanoparticles to the core of the tumors, and the other is poor retention of nanoparticle in the cytoplasma. Our latest study reported a multicomponent-based microemulsion composed of coix seed oil (an approved anticancer agent in China), ginsenoside Rh2 (G-Rh2, a drug resistance modulator with anticancer capability) and etoposide (a potent chemotherapeutic agent) (ECG-MEs), which was capable of synergistically treating the drug-resistant breast cancer through rational drug combination and sequential release that prolonged the retention within the tumor cells (Qu et al., 2017). However, whether ECG-MEs possess the similar anticancer effects against other types of cancer (such as non-small cell lung cancer) is still unclear. In addition, we previously found that different ratios of G-Rh2 directly influenced the size of ECG-MEs, but such effects on the architecture of microemulsion and delivery efficiency within the tumor tissue also need to be further elucidated.

Herein, we developed ECG-MEs based on the previous method to treat the A549 (human lung cancer cells) tumor-bearing mice. This study focused on the optimization of preparation, characterization of the microemulsions, validation of the synergistic anticancer effect, and evaluation on A549 cellular uptake. Of note, we fabricated ECG-MEs with two different particle sizes to investigate the penetration on 3D tumor sphere model. Furthermore, the anticancer efficacy, safety *in vivo* and the relative molecular mechanisms were also performed.

## Materials and methods

### Materials

Coix seed oil was extracted from *Coix Lacryma-jobi* by our group (Qu et al., 2014), the purity was determined as 87.6% using glycerin trilaurate as a reference. G-Rh2 (purity >98%) was purchased from Nanjing Zelang BioTECH Co. (Jiangsu, China). Etoposide (purity >99.9%) was bought from Sigma-Aldrich Co., Ltd. (Poole, UK). Cremophor^®^ RH40 was provided by BASF Co., Ltd. (Ludwigshafen, Germany). PEG400, amiloride, genistein, chlorpromazine, and ammonia chloride were purchased from Sinopharm Chemical Reagent Co., Ltd. (Shanghai, China). Roswell park memorial institute (RPMI) 1640 medium, fetal bovine serum (FBS), penicillin–streptomycin solution, MTT cell proliferation and cytotoxicity detection kit were bought from KeyGEN BioTECH Co., Ltd. (Jiangsu, China). The fluorescence probes including DiI and fluorescein isothiocyanate (FITC) were purchased from Beyotime Biotech Co., Ltd. (Shanghai, China). The water used in this study was produced by Elix^®^5 Milli Q-water purification system (Millipore, Billerica, MA). The other chemical reagents were of analytical grade, unless otherwise statement.

### Animals

Male BALB/c (nu/nu) nude mice with the average weight of 21 g were purchased from Changzhou Cavens Laboratory Animal Co. Ltd. (Jiangsu, China). The animals were ad libitum fed food and water. The operations to animal model including tumor xenograft implantation, intraperitoneal injection, blood collection, and organ harvest were in accordance with the Guidelines for Care and Use of Laboratory Animals, approved by the Animal Experimentation Ethics Committee of Nanjing University of Chinese Medicine (Jiangsu, China).

### Optimization of preparation of ECG-MEs

In our previous study, the preparation technology of ECG-MEs had been reported as a ‘one-step emulsion’ method (Qu et al., 2015, 2017). Hereon, we plotted pseudoternary phase diagram to further investigate the influence of weight ratio between G-Rh2, RH40 and PEG400 on the formation of microemulsion. The specific experiment process is as follows. First, RH40 and G-Rh2 at the weight ratio of 11/1 ∼ 13/1 were used as the mixed surfactant, and then mixed with various amounts of PEG400 and 400 mg of coix oil at three different K_m_ (the weight ratio of mixed surfactant to PEG400) values (1/1, 2/1, and 3/1). Next, the resulting mixture was vigorously stirred at 45 °C until the above-mentioned components became completely homogeneous. At the end of this time, 100 μL aliquots of deionized water was slowly added into the mixture. The microemulsion region was circled in the pseudoternary phase diagram after connecting the points representing a nano-sized emulsion with the characteristics of transmittance, clarity, and water-like fluidity.

### Preparation and characterizations of ECG-MEs

Previous preparation of ECG-MEs (Qu et al., 2017) was moderately modified in this study. Four mg of etoposide, 400 mg of coix seed oil, 360 mg of RH40, 120 mg PEG400 and 30 mg of G-Rh2 were synchronously mixed at 45 °C using a mechanical stirrer. After 2 h of stirring, 3.0 mL of deionized water was added into the resulting mixture to obtain a clear and transparent ECG-MEs solution. In addition, etoposide-loaded coix seed oil microemulsion (EC-MEs) and G-Rh2-loaded coix oil microemulsion (GC-MEs) as the control formulations, was synthesized through the similar preparation of ECG-MEs, but without G-Rh2 or etoposide, respectively. The drug encapsulation efficiency (EE) of ECG-MEs involving the quantification of etoposide and G-Rh2, and relative manipulation was referred by the previous publications (Qu et al., 2017). The essential parameters of microemulsion characterization, such as particle size, polydispersity index (PDI) and surface potential were measured by dynamic light scattering (DLS) laser particle size analyzer (Nano ZS; Malvern, UK). The morphology of microemulsions was observed by transmission electron microscopy (TEM). Prior to the experiment, the freshly prepared microemulsions with a coix oil concentration of 80 mg/mL were dispersed onto a TEM-exclusive copper grid, followed by staining with 10 μL of phosphotungstic acid solution (1%, v%). The microemulsion was immediately observed by TEM (JEM-200CX, JEOL, Tokyo, Japan) after drying under an infrared lamp.

### Cell culture

The human non-small lung cancer cells (A549) and green fluorescence protein-transfected A549 (GFP/A549) cells were cultured in RPMI 1640 medium, which was additionally added FBS (10%, v%), penicillin (100 IU/mL) and streptomycin (100 μg/mL), under an atmosphere of 5% CO_2_ and 90% relative humidity. The culture medium was replaced every two days during the cell culture.

### Antiproliferative effect (Tong et al., 2017)

Five thousand of A549 cells were seeded in a well of 96-well plates for 24 h. The complete RPMI 1640 medium was replaced with 200 μL of different formulations. After treatment for 48 h, the cells were stained with 20 μL of the MTT (5 mg/mL) for 4 h, followed by removing the medium of each well, and dissolving the resulting formazan crystals by 200 μL of dimethyl sulfoxide (DMSO). The absorbance was detected at 570 nm using the microplate reader (Varioskan Flash, Thermo). The cell viability and combined index (CI) were calculated by the following formulas, respectively. Viability (%) = absorbance of sample/absorbance of control; CI = IC_50_^a^/IC_50_^A ^+IC_50_^b^/IC_50_^B ^+IC_50_^c^/IC_50_^C^, IC_50_^a^, IC_50_^b^, and IC_50_^c^ represent the half-maximal inhibitory concentration of each component in combination formulation, respectively; IC_50_^A^, IC_50_^B^, and IC_50_^C^ represent the half-maximal inhibitory concentration of single component, respectively (Zhong et al., 2017).

### Cell apoptosis induction

The manipulate procedure was similar to our previous works (Qu et al., 2014; Li et al., 2017). Briefly, the adherent A549 cells with 80% overspread were treated with different formulations for 5 h, followed by trypsinization and incubation with the equivalent volume of Annexin V-PE/7-AAD apoptosis detection kit (Guava, Merck Millipore, Billerica, MA) for 15 min in the dark. At the end of this time, the stained cells were immediately analyzed by the flow cytometry (Guava 6HT, Merck Millipore, Billerica, MA).

### Cellular uptake and mechanism exploration

In this experiment, FITC-labeled ECG-MEs (FITC/ECG-MEs) was prepared to evaluate the intracellular accumulation of formulations. One hundred thousand of A549 cells were seeded in each well of 6-well plates and rinsed with cold phosphate buffer saline (PBS) thrice, followed by incubation with 1 mL of FITC/ECG-MEs containing 5 μM of FITC at 37 °C for 6 h. Afterward, the cells were rinsed with cold PBS thrice, and then analyzed using fluorescence inverted microscope (Olympus, IX73) and flow cytometry, respectively. Likewise, the competitive inhibition was evaluated through pre-treating A549 cells with low temperature or specific endocytosis inhibitors (Su et al., 2014; Matsubara et al., 2017), such as ammonia chloride (NH_4_Cl), genistein (Gen), chlorpromazine (Chlo) and amiloride (Amilo), for 1 h and subsequently incubated with FITC/ECG-MEs for 4 h in the presence of the above-mentioned inhibitors. The intracellular fluorescence was measured by flow cytometry after washing with 2 mL of ice-cold PBS thrice. In addition, FITC, the mixture of FITC, etoposide, coix seed oil and G-Rh2 (mixed FITC/ECG), and FITC-labeled EC-MEs (FITC/EC-MEs) were employed as the controls.

### Construction for A549 three-dimensional (3D) tumor spheroids

In order to develop the 3D tumor spheroids with green fluorescence, we used GFP/A549 cells to construct model by the previously reported methods but with some modifications (Ju et al., 2014). In brief, 0.1 mL of sterile RPIM 1640 culture medium containing 2% (wt%) agarose was pre-coated in each well of 96-well plates at 80 °C. After cooling to the room temperature, 5 × 10^3^ of GFP/A549 cells were seeded on the surface of the agarose-based culture medium, followed by shaking for 5 min. After incubation in the cell incubator for 5–7 d, the 3D tumor spheroids model with the diameter about 700 μm were selected by fluorescence inverted microscope and used in the penetration studies.

### *In vitro* penetration of microemulsion

To visualize the penetration of microemulsions within the tumor spheroids, red fluorescence DiI labeled ECG-MEs (DiI/ECG-MEs) were prepared by the similar method of ECG-MEs. Prior to the experiment, the tumor spheroids with compact structure and uniform shape were carefully transferred to a confocal glass bottom dish. And then, DiI/ECG-MEs (DiI concentration, 2.5 μM) with two different particle sizes were incubated with tumor spheroids for 8 h at 37 °C. Next, the tumor spheroids were rinsed with PBS, fixed with 4% (v%) paraformaldehyde, and observed by confocal laser scanning microscope (CLSM) with Z-stack imaging technology (10 μm intervals from the surface to the middle), successively.

### *In vivo* antitumor efficacy

The BALB/c (nu/nu) nude mice weighed around 21 ± 2 g were randomly divided into five groups (*n* = 8). After 7 d of adaptive feeding, 2 × 10^7^ A549 cells were subcutaneously injected into the right back of mice to establish the A549 tumor bearing mice models. When the average tumor size reached approximately 100 mm^3^, mice in each group were treated (intragastric administration) with ECG-MEs, etoposide, EC-MEs, and ECG mixture with an etoposide dosage of 12 mg/kg once daily for two weeks. Saline was employed as the negative control. At day 24, after 72 h of the therapeutic endpoint, the mice were euthanized, followed by collecting blood, tumors and the normal organs. Thereafter, the formalin-fixed organs were embedded in paraffin blocks and then prepared to pathological section for hematoxylin and eosin (HE) and terminal deoxynucleotidyl transferase dUTP nick end labeling (TUNEL) staining (KeyGen Biotech, Jiangsu, China). All the experiments were in accordance to the manufacturer’s protocol and the images of sections were observed by the fluorescence-inverted microscope.

### Systemic toxicity and cytokines detection

The *ex vivo* liver and kidney were weighed to calculate the liver and kidney indices according to the previously reported formulas (Huo et al., 2017). An amount of 100 μL of blood samples of the mice treated with various formulations were collected for blood routine, liver function, kidney function, and cytokines analysis. For evaluation on the toxicity against liver and kidney, 200 μL of serum was prepared to detect the concentration of aspartate transaminase (AST), alanine transaminase (ALT), and blood urea nitrogen (BUN) at day 24. Furthermore, different types of cytokine reflecting the influence of treatment on immune functions, such as interferon-γ (IFN-γ), interleukin-2 (IL-2), interleukin-12α (IL-12α), interleukin-6 (IL-6), interleukin-10 (IL-10), monocyte chemoattractant protein-1 (MCP-1), transforming growth factor β1 (TGF-β1), and tumor necrosis factor-α (TNF-α) were quantified in accordance to the manufacturer’s protocol of respective enzyme-linked immunosorbent assay (ELISA) kits (Abcam, Cambridge, UK).

### Data analysis

All the data given in this study are mean ± standard deviation. Statistical test was performed through two-tailed Student’s *t* test. *p < .05 and **p < .01 represented the significant and extremely significant difference, respectively.

## Results and discussion

### Pseudoternary phase diagrams plotting

**Figure 1.**
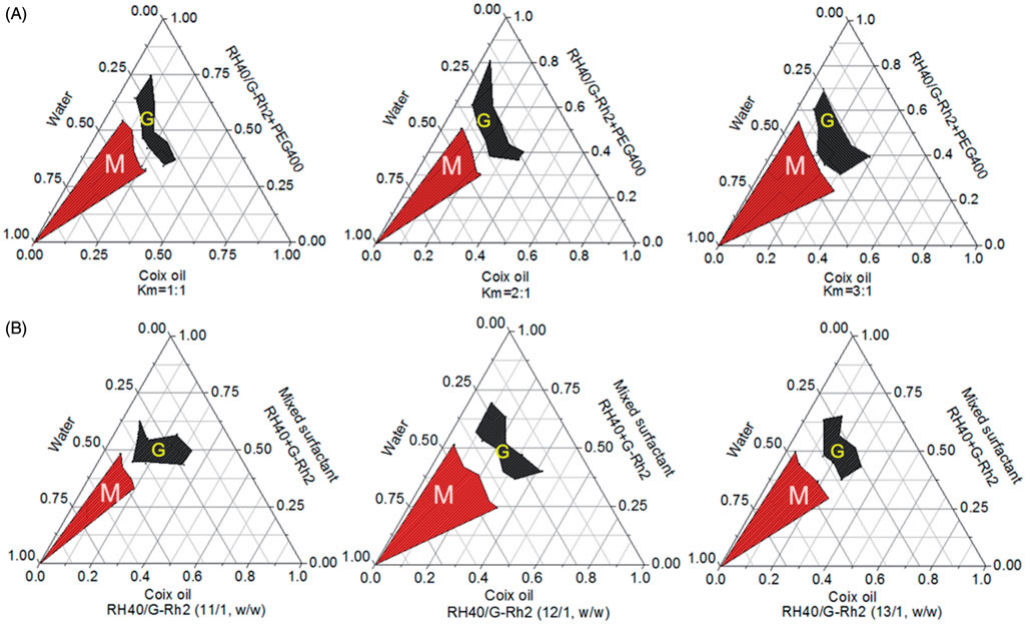
Optimization for the preparation of microemulsion composed of coix oil, mixed surfactant (RH40 + G-Rh2) and PEG400 at (A) different K_m_ values and (B) various weight ratios of RH40 to G-Rh2 through plotting the pseudoternary phase diagrams. The red region (M) and the gray region (G) represent the microemulsion and gel formulation under the corresponding preparation condition, respectively.

Pseudoternary phase diagram is a useful tool to evaluate the capability of microemulsion formation through comparing the microemulsion area to others (Yehia et al., 2017). Previously, we have validated that coix oil microemulsion with the weight ratio of RH40 to PEG400 (K_m_) at 2:1 received the largest area of microemulsion. In addition to conventional surfactant RH40, G-Rh2 as a functional surfactant was also employed to assemble multicomponent microemulsion in this study. Herein, the optimal K_m_ value was further determined through adjusting the weight ratio of mixed surfactant (RH40/G-Rh2, 12/1, w/w) to PEG 400. As shown in Figure 1(A), ECG-MEs possessed the largest microemulsion formation area when the K_m_ value was determined as 3/1, suggesting that preparation technology with such K_m_ value could readily form a clear and transparent microemulsion. Likewise, the weight ratio of RH40 to G-Rh2 was also optimized when we set K_m_ value as 3/1. As shown in Figure 1(B), the zone representing microemulsion formation was the largest when the weight ratio of RH40 to G-Rh2 was determined as 12/1. Considering the limited amphipathy, either excessive or insufficient G-Rh2 probably brought about the problems of systemic compatibility, and thereby impeding the assembly of microemulsion. Based on our obtained results (Qu et al., 2017), the optimal weight ratio between coix oil, RH40, G-Rh2, PEG400 and etoposide was determined as 200/180/15/60/2 in this study.

### Characterizations of various microemulsions

**Figure 2.**
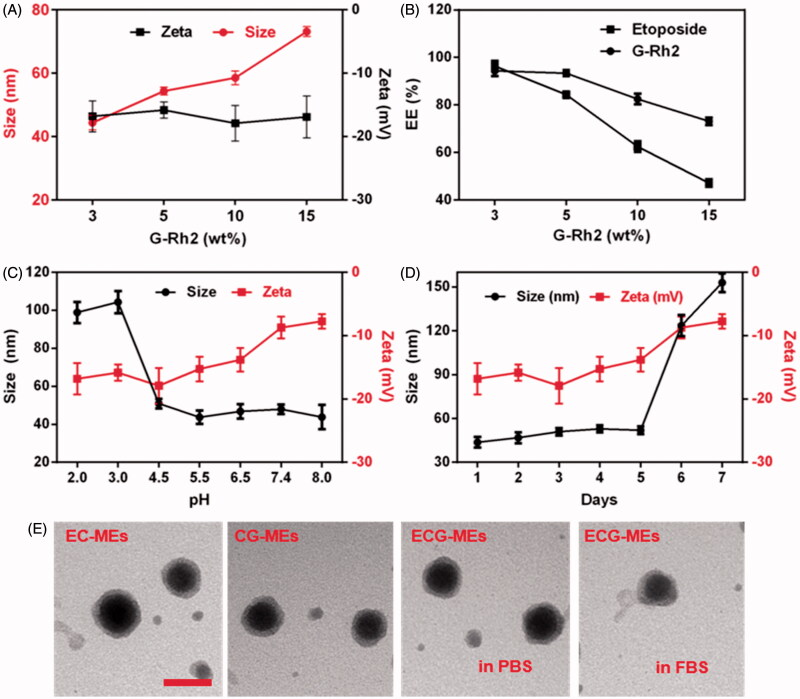
Characterizations of microemulsions. (A) The particle size and zeta potential of ECG-MEs with the different amounts of G-Rh2. All the data are presented as mean ± SD (*n* = 3). (B) The drug encapsulated efficiency (EE) of ECG-MEs with different amounts of G-Rh2. All the data are presented as mean ± SD (*n* = 3). The changes of size and zeta potential of ECG-MEs under (C) different pH values and (D) within 7 d. All the data are presented as mean ± SD (*n* = 4). (E) TEM images of various types of microemulsions. The scale bar is 50 nm.

Rational combination of anticancer drugs, optimal proportion of each component and high *in vivo* stability are of great importance to multicomponent-based microemulsion for synergistic antitumor efficacy. As shown in Figure 2(A), the particle size of ECG-MEs significantly increased from 44.3 to 73.1 nm with the content of G-Rh2 raised, but the zeta potential had no obvious variety even after incorporation of excessive G-Rh2, suggesting that overloading of G-Rh2 might lead to the increase in the average particle size. The EE of etoposide and G-Rh2 of ECG-MEs with 3% (wt%) G-Rh2 were 96.3 and 94.3%, respectively. However, approximately 40 and 50% of etoposide were leaked from ECG-MEs with the content of G-Rh2 increased up to 10 and 15%, respectively (Figure 2(B)). Likewise, overloading of G-Rh2 also impeded the encapsulation of etoposide, probably because substantial G-Rh2 competitively occupied the hydrophobic core of microemulsion. The stability of microemulsion was investigated under different pH environments and storage time. As shown in Figure 2(C), 0.5 h of incubation under different pH values ranged from 4.5 to 8.0 did not lead to notable alternation in the particle size of ECG-MEs. In comparison, the size sharply increased up to 100 nm with the pH value reduced below 3.0, indicating an acid-induced drug release in the stomach (Zhang et al., 2017). Besides, ECG-MEs was capable of storing stably within 5 d at the room temperature (Figure 2(D)). Of note, temperature lower than 10 °C might bring about the considerable leakage of etoposide from ECG-MEs (Figure S1), probably because of the low freezing point of RH40, and such process was irreversible even the microemulsions were warmed back to the room temperature. To evaluate the influence of each component on the morphology of microemulsion, the TEM images of EC-MEs, CG-MEs, and ECG-MEs in PBS and 50% FBS solution were displayed in Figure 2(E). No notable difference of size and shape was found among all the samples. These results suggested that ECG-MEs with 3% G-Rh2 had a small size, high drug encapsulation efficiency and acceptable stability under various environments.

### Cellular studies

#### Internalization of microemulsions

**Figure 3.**
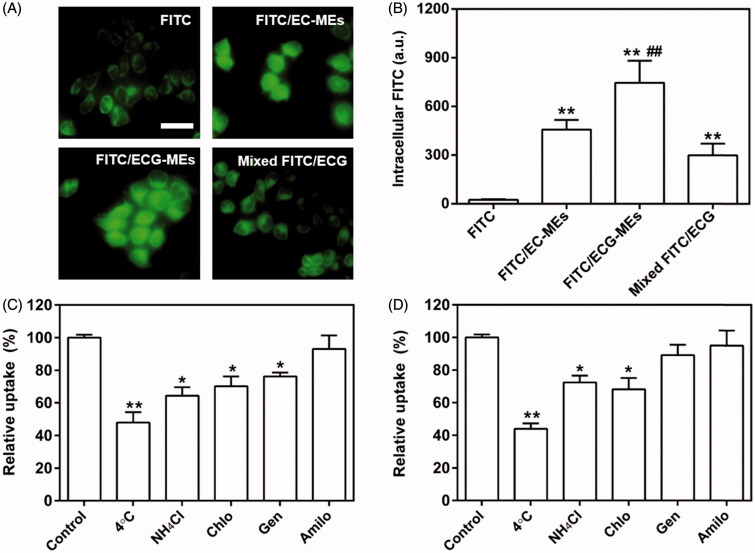
Cellular studies. (A) The fluorescence images of A549 cells treated with various formulations observed by the fluorescence-inverted microscope. The scale bar is 50 μm. (B) Quantitation of intracellular green fluorescence intensity measured using the flow cytometry. **p < .01 versus FITC; ##*p* < .01 versus FITC/EC-MEs. All the data are presented as mean ± SD (*n* = 3). (C,D) Mechanism of cellular uptake. The relative uptake efficiency of (C) ECG-MEs and (D) EC-MEs after pretreating A549 cells with different endocytosis inhibitors. **p < .01 versus control. All the data are presented as mean ± SD (*n* = 4).

To reveal the influence of G-Rh2 and microemulsion formulation on cellular uptake, we investigated the intracellular fluorescence of A549 cells treated with FITC/ECG-MEs, FITC/EC-MEs, mixed FITC/ECG and free FITC, respectively. As shown in Figure 3(A,B), the fluorescence of FITC/ECG-MEs was significantly higher than that of FITC/EC-MEs, suggesting that G-Rh2 was favorable to the intracellular accumulation of microemulsions. We have previously validated that G-Rh2 was able to enhance the internalization of ECG-MEs on multidrug resistant breast cancer cells. Taken the obtained results together, ECG-MEs might be capable of entering various types of tumor cells efficiently. In addition, the cellular uptake of FITC/EC-MEs was greatly stronger than free FITC, indicating a native advantage of microemulsion formulation on crossing the cell membrane. However, no obvious difference between FITC/EC-MEs and mixed FITC/ECG was observed, suggesting that microemulsion formulation and G-Rh2 cooperatively promoted the intracellular distribution of anticancer components.

#### Mechanism of cellular uptake

To dissect the mechanism of A549 cellular uptake, several endocytosis pathways were blocked in advance by low temperature and specific uptake inhibitors. As shown in Figure 3(C), the incubation at 4 °C remarkably suppressed the internalization of ECG-MEs. It suggested that the endocytosis of ECG-MEs was in an energy-dependent manner (Matsubara et al., 2017). Furthermore, the interventions of ammonia chloride, genistein, and chlorpromazine could also significantly reduce the cellular uptake of FITC/ECG-MEs, indicating that microemulsions entered the cells via the clathrin-mediated and caveolae-mediated cotransport pathways. In contrast, genistein did not decrease the intracellular accumulation of EC-MEs (Figure 3(D)). The obtained results meant that the presence of G-Rh2 was able to avert the endo/lysosomal distribution of microemulsion, increase drug release in the cytoplasma, and thereby enhancement on anticancer capability.

### *In vitro* tumor penetration

**Figure 4.**
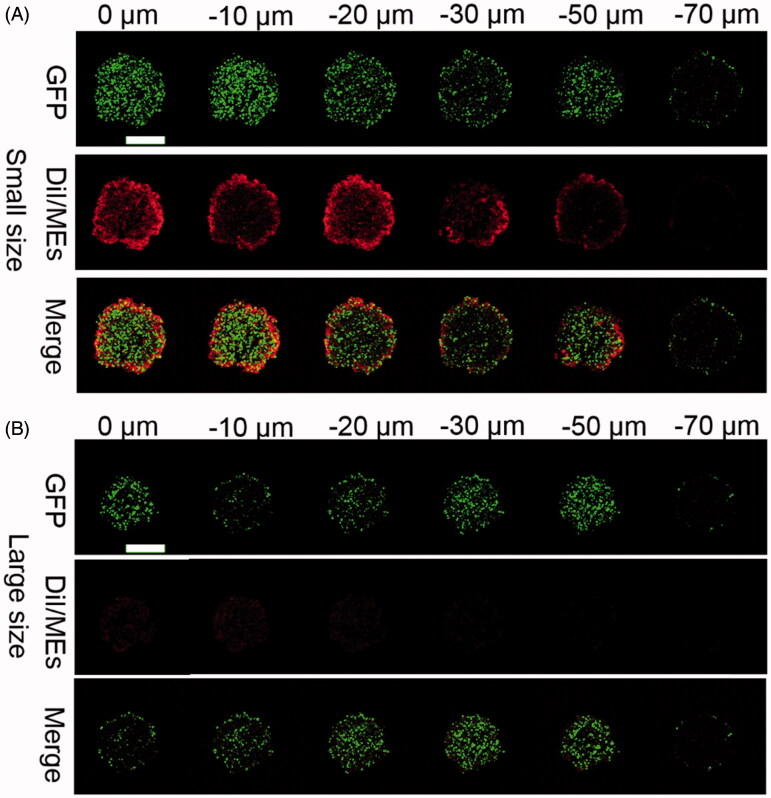
Evaluation on the penetration of microemulsion with (A) small size (40 nm) and (B) large size (120 nm) using A549 3D tumor spheres model. The CLSM images were used Z-stack imaging technology with 10 μm intervals from the surface to the core. The green represents the autofluorescence of GFP-transfected A549 cells and the red represents the penetrated DiI/MEs. The scale bar is 500 μm.

Deep tumor penetration is a factor of great concern for nanoparticles to deliver the payload to the deep layer of the tumor tissues (Heldin et al., 2004), but still faces a big challenge because of several well-known reasons, such as abnormal tumoral vasculture, denser fiber network in the extracellular matrix (ECM), lymphatic drainage dysfunction, and so on (Carmeliet & Jain, 2000; Leu et al., 2000; Netti et al., 2000). Generally, nanoparticles with small size had a more potent tumor penetration than the larger counterparts (Wilhelm et al., 2016). To verify the influence of particle size on the deep tumor penetration, we fabricated DiI-labeled ECG-MEs (DiI/ECG-MEs) with two different sizes (40 and 120 nm) through adjusting the content of G-Rh2. As shown in Figure 4, the green fluorescence represented the 3D GFP-transfected A549 tumor sphere, and the red fluorescence reflected the intratumoral distribution of DiI/ECG-MEs. We have clearly observed that DiI/ECG-MEs with a small size spread from the surface of tumor sphere to 60 μm in depth after treatment for 12 h. In contrast, only the weak fluorescence was found at the periphery of the tumor spheroids after treatment with large-sized DiI/ECG-MEs. It indicated that 3% of G-Rh2 is not only capable of maintaining a stable nanostructure for ECG-MEs, but also favorable to a small size-mediated deep penetration, and thereby a potential improvement on anticancer efficacy.

### Cytotoxicity and apoptosis induction

**Figure 5.**
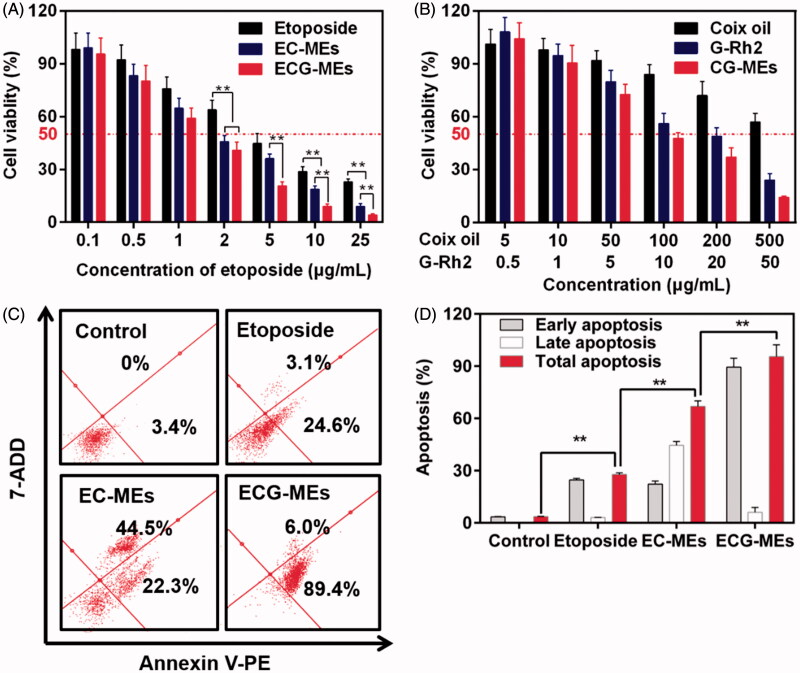
(A,B) Cytotoxicity of different treatments against A549 cells for 48 h. **p < .01, all the data are presented as mean ± SD (*n* = 6). (C,D) A549 cell apoptosis induced by different etoposide formulations. (C) In each quadrant of apoptosis, the upper right and lower right zones represent the late apoptosis and the early apoptosis, respectively. (D) The quantification of cell apoptosis rate after various treatments. **p < .01, all the data are presented as mean ± SD (*n* = 4).

We have previously verified that ECG-MEs had the great advantages in the treatment of multidrug resistant breast cancer via the G-Rh2-mediated P-gp inhibition. To explore whether multicomponent combination could also realize the synergistic antitumor effect against A549 cells, the cytotoxicity of EC-MEs, CG-MEs, and ECG-MEs were investigated by the classic MTT method. As shown in Figure 5(A) and Table 1, the IC_50_ and CI of ECG-MEs were 1.49 μg/mL and 0.70, respectively, exhibiting the strongest cytotoxicity and the lowest CI among all the test groups. It was attributed to the rational drug combination and the enhanced drug distribution in cytoplasma resulted from the incorporation of G-Rh2. However, there was no obvious synergistic effect after the treatment of CG-MEs (Figure 5(B)). It suggested that the main contribution to the cell killing activity of ECG-MEs was from etoposide, which probably received assistances from other components to amplify the antitumor effects. Logically, 95.4% of A549 cells were induced to apoptosis after treatment with ECG-MEs for 5 h, which displayed an overwhelming advantage over EC-MEs and etoposide (Figure 5(C,D)). These results could be explained by the enhanced cellular uptake, reduced endo/lysomal entrapment and consequently promoted inhibition of type II topoisomerase.

**Table 1.** The IC_50_ and combined index of various types of formulations against A549 cells (*n* = 6).

Formulation	IC_50_^e^ (μg/mL)	IC_50_^c^ (μg/mL)	IC_50_^g^ (μg/mL)	CI
Etoposide	5.03	NA	NA	NA
Coix oil	NA	344.78	NA	NA
G-Rh2	NA	NA	16.42	NA
CG-MEs	NA	139.91	13.92	1.26
EC-MEs	2.64	79.26	NA	0.75
ECG-MEs	1.49	44.72	4.47	0.70

IC_50_^e^ represents the half-maximal inhibitory concentration of etoposide; IC_50_^c^ represents the half-maximal inhibitory concentration of coix oil; IC_50_^g^ represents the half-maximal inhibitory concentration of G-Rh2; and CI represents the combined index.

### *In vivo* antitumor efficacy

**Figure 6.**
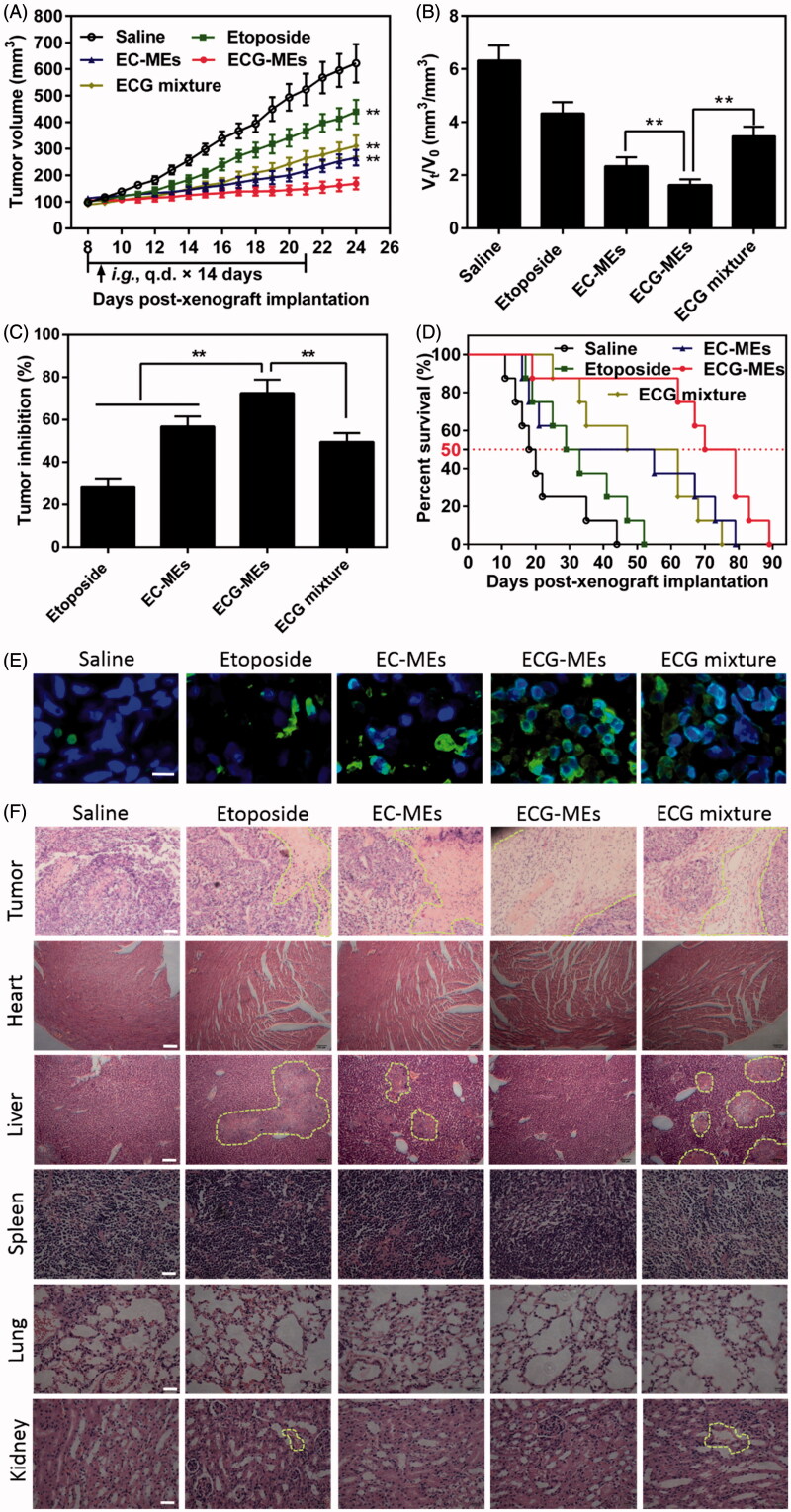
Evaluation on the *in vivo* antitumor efficacy. (A) The changes in the tumor growth of A549 tumor-bearing mice after various treatments for two weeks. The arrow represents the time of intragastric administration. **p < .01 versus ECG-MEs. All the data are presented as mean ± SD (*n* = 8). (B) The ratio of V_t_ to V_0_, V_0_, and V_t_ represent the volume of tumors at day 8 and day 24, respectively. **p < .01, all the data are presented as mean ± SD (*n* = 8). (C) The inhibition rate of tumor growth under different treatments. **p < .01, all the data are presented as mean ± SD (*n* = 8). (D) Kaplan–Meier survival curves of the A549 tumor-bearing mice within 90 d. (E) The tumor sections stained with FITC-dUTP using fluorescence inverted microscope. The green represents the DNA fragments of apoptotic cells. The blue represents the nuclei. The scale bar is 20 μm. (F) The images of different organ sections stained with HE after various treatments. The scale bar is 100 μm.

To validate the advantages of integrating G-Rh2 into EC-MEs system, the antitumor efficacy was performed through intragastrical administration of ECG-MEs on the A549 tumor-bearing mice, with saline, etoposide, EC-MEs and ECG mixture as the controls. As shown in Figure 6(A), the treatment of EC-MEs exhibited an obvious inhibition of tumor growth in comparison to that of etoposide, which could be explained by an improved tumorous accumulation based on the nanoparticle-mediated EPR effect. With the incorporation of G-Rh2, ECG-MEs further retarded the tumor growth compared to EC-MEs, highlighting that G-Rh2 facilitated a prolonged retention within the tumor cells and a more efficiently synergistic antitumor effect with other components. Furthermore, the antitumor capability of ECG mixture was significantly weaker than that of ECG-MEs. These results indicate that rational combination and stable codelivery of multicomponent are two indispensable factors for enhanced anti-lung cancer efficacy *in vivo*. At the end of the observation, the tumor size of mice at day 8 and day 24 was respectively measured to evaluate the increment rate, which was another index to reflect the inhibition of tumor growth. As shown in Figure 6(B), the tumor size in saline, etoposide, and EC-MEs groups increased by 6.3, 4.3, and 2.4-fold during the treatments, respectively. By comparison, the tumor size only grew 1.6-fold after treatment with ECG-MEs. In addition, the inhibition of tumor growth of ECG-MEs treatment reached 72.56 ± 6.31%, which was the highest among all the test groups (Figure 6(C)), further verifying the potent antitumor ability against A549 tumor-bearing mice model. Besides, Kaplan–Meier survival curves of animals were plotted to investigate whether the improved pharmacodynamics bring about the prolonged survival time. As displayed in Figure 6(D), the survival time and median survival time of mice treated with ECG-MEs were 70 and 89 d, respectively, which were obviously longer than other treatments. The TUNEL staining (Figure 6(E)) showed that ECG-MEs-treated tumor tissue was subjected to the most obviously apoptotic induction, as evidenced by the overwhelming green-labeled DNA fragment. Likewise, the massive abnormality of nuclear morphology (yellow dotted line-circled region) was observed in the HE staining image of tumor tissue of mice treated with ECG-MEs (Figure 6(F)). Taken together, all the obtained results validated that ECG-MEs were capable of inhibiting the tumor growth and prolonging the survival time via multicomponent-based synergistic antitumor capability and small size-mediated deep tumor penetration.

### Evaluation on safety and cytokines

**Figure 7.**
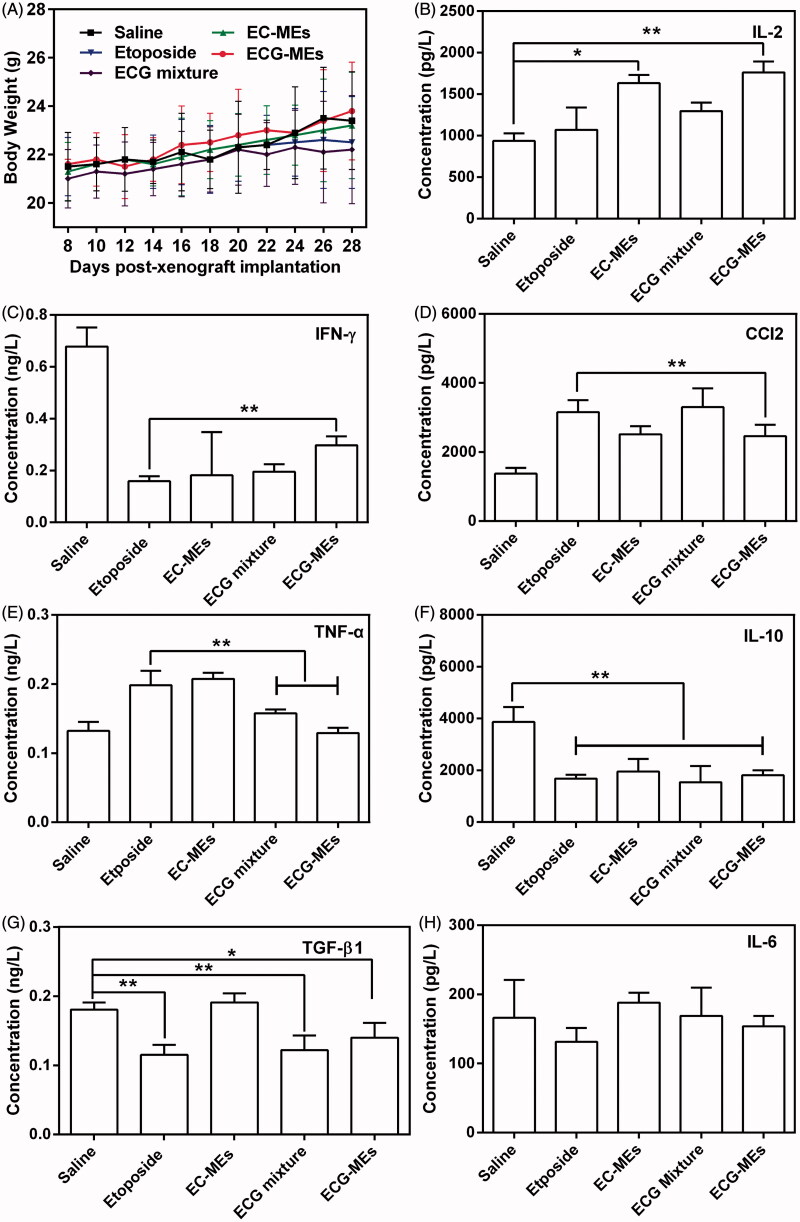
Evaluation on safety. (A) The changes in the body weight of mice during the treatment. All the data are presented as mean ± SD (*n* = 8). (B–H) Cytokine detections. The content of (B) IL-2, (C) IFN-γ, (D) CCl2, (E) TNF-α, (F) IL-10, (G) TGF-β1, and (H) IL-6 in the serum of mice after 72 h of the last administration. **p < .01, all the data are presented as mean ± SD (*n* = 8).

According to the previous reports, orally etoposide formulation might result in severe acute damages at liver and kidney (Mukherjee et al., 2011) because of nonselective biodistribution. In this study, the body weight and HE staining of the main normal organs were monitored during and after the treatments, respectively. As shown in Figure 7(A), the body weight of mice in each group had no statistically difference during the observing period, indicating a negligible potential systemic toxicity at the determined dose. Additionally, HE-stained pathological section results showed that the massive cell necrosis or inflammation occurred in liver tissues of mice treated with etoposide and ECG mixture (Figure 6(F)). By comparison, such damage was significantly mitigated after administration of EC-MEs, and further reduced with the treatment of ECG-MEs, indicating a feasibility of multicomponent combination in reducing the side effects (Figure 6(F)). Furthermore, the mild kidney toxicity resulted from the treatments of etoposide and ECG mixture was not found in EC-MEs and ECG-MEs groups. In addition to pathological sections, the liver and spleen index, AST, ALT, and BUN were also performed to further prove the high safety of ECG-MEs treatment. As exhibited in Table 2, etoposide resulted in a significantly reduced liver index and the obviously increase in liver enzymes (AST and ALT). However, such side effects were not observed in ECG-MEs-treated group, suggesting that combination therapy and microemulison formation could alleviate the chemotherapy-associated systemic toxicity.

**Table 2.** The weight ratio of liver/spleen to the body and the serum concentration of liver enzymes and acute renal lesion indicator.

Formulation	Liver index (mg/g)	Spleen index (mg/g)	ALT (IU/mL)	AST (IU/mL)	BUN (mmol/L)
Saline	54.28 ± 4.73	6.24 ± 0.42	49.6 ± 5.33	198.3 ± 11.17	12.54 ± 2.56
Etoposide	45.24 ± 3.17*	6.03 ± 0.58	82.5 ± 9.57**	273.4 ± 27.68**	13.45 ± 1.37
EC-MEs	49.64 ± 3.29	5.98 ± 0.62	68.4 ± 6.72	168.2 ± 24.36	10.24 ± 1.01
ECG-MEs	52.62 ± 2.92	6.37 ± 0.44	59.5 ± 6.80	210.6 ± 14.24	11.62 ± 1.27
ECG mixture	48.26 ± 5.25	6.18 ± 0.34	79.5 ± 4.19**	292.44 ± 32.24**	15.24 ± 4.53

*p < .05; **p < .01 versus saline, all the data are presented as mean ± SD (*n* = 8).

In order to evaluate etoposide-caused immune suppression and potential inflammatory response, we measured the serum content of several cytokines after 72 h of the last administration. Generally, T cells-secreted cytokines were categorized into T helper 1 (Th1) and T helper 2 (Th2). The former type such as IFN-γ and IL-2 is favorable to elicit antitumor immunity, and the latter one typified by IL-6, IL-10, MCP-1, TGF-β1, and TNF-α inactivate the above-mentioned immunity and consequently induce the inflammation responses (Huo et al., 2017). Therefore, antitumor researchers have paid more attention in how to induce the transformation of Th2 into Th1 for yielding the enhancement on antitumor efficacy. As shown in Figure 7(B), ECG-MEs significantly increased the level of IL-2 compared with etoposide, indicating a promotion of antitumor potentials given by the combination of etoposide, coix oil, and G-Rh2. As for the expression of IFN-γ, ECG-MEs gained an obvious improvement in comparison of etoposide, although all the treatments were greatly lower than saline group (Figure 7(C)), suggesting that ECG-MEs was capable of alleviating the suppression of IFN-γ induced by etoposide. In addition, the serum concentration of CCl2 and TNF-α in ECG-MEs group dramatically reduced than etoposide (Figure 7(D,E)). Furthermore, ECG-MEs significantly decreased the concentration of IL-10 and TGF-β1 in comparison to negative control (Figure 7(F,G)), reflecting the obvious alleviation of inflammation *in vivo*. There was no obvious variety of IL-6 content in all the groups (Figure 7(H)). Taken together, elevating Th1 and suppressing Th2 were highly relative to improvement of ECG-MEs treatment on antitumor efficacy.

## Conclusion

In this study, we developed a multicomponent-based microemulsion combining the advantages of etoposide, coix seed oil, and G-Rh2 for anti-lung cancer treatment. The weight ratio of G-Rh2 was optimized by multidimensional characterizations. Owing to the rational drug combination, ECG-MEs displayed the small particle size, high drug encapsulation efficiency and good stability under different physiological environments. In the A549 cells and xenograft tumors, ECG-MEs exhibited the obvious synergistic effect on cytotoxicity, apoptosis induction, inhibition of tumor growth and survival time. Intriguingly, such improvement on anticancer efficacy was attributed to the prolonged retention in cytoplasma, deep tumor penetration, and increase of Th1 cytokines. We believe that ECG-MEs promises to be an efficient drug delivery for the anti-lung cancer treatment.

## Supplementary Material

IDRD_Ding_et_al_Supplemental_Content.docx
