# Supplementary material for: A multicomponent microemulsion using rational combination strategy improves lung cancer treatment through synergistic effects and deep tumor penetration
Source: Drug Deliv. 2017 Aug 25;24(1):1179–90. doi: 10.1080/10717544.2017.1365394 (PMC8241011; doi:10.1080/10717544.2017.1365394)
Supplement: IDRD_Ding_et_al_Supplemental_Content.docx [file IDRD_A_1365394_SM4197.docx]

Figure S1. The influence of temperature on the particle size and encapsulation of ECG-MEs. All the data are presented as mean ± SD (n = 3).
